# Fear Of cancer Recurrence Group-based intervEntion (FORGE): Study protocol for the co-design of a psychological intervention for young adult cancer survivors

**DOI:** 10.12688/hrbopenres.14446.4

**Published:** 2026-11-12

**Authors:** Catherine O'Brien, Maria Pertl, Emer Guinan, Andrew Darley, Suzanne Guerin, Sonya Collier, Maeve Lowery

**Affiliations:** 1 Trinity College Dublin School of Medicine, Dublin, Leinster, Ireland; 2 St James's Hospital, Dublin, Leinster, Ireland; 3 Royal College of Surgeons in Ireland Department of Psychology, Dublin, Leinster, Ireland; 4 University College Dublin School of Nursing Midwifery and Health Systems, Dublin, Leinster, Ireland; 5 University College Dublin School of Psychology, Dublin, Leinster, Ireland

**Keywords:** Fear of Cancer Recurrence, Co-design, Group-based intervention, Public and Patient Involvement, Psychological intervention, Cancer survivor

## Abstract

**Background:**

Fear of cancer recurrence (FCR) represents one of the most frequently reported unmet needs among cancer survivors, with young adults demonstrating heightened susceptibility to its effects. Although a range of specialist-delivered psychological interventions has shown efficacy in managing FCR across adult populations, much of this evidence is derived from one-to-one therapeutic formats. Adapting such interventions to a group setting, both to enhance peer support and to improve resource efficiency, necessitates careful adaptation to ensure the programme is appropriately tailored to the group context. The aim of this study is to co-design the adaptation of an existing one-to-one FCR psychological intervention to a group-based setting to support psychosocial distress across the spectrum of FCR needs of young adult cancer survivors in Ireland.

**Methods:**

This co-design study will use the ADAPT framework to conduct a series of workshops that adopt a modified Nominal Group Technique (NGT) model. Two groups of co-design stakeholders will be recruited nationally (i) *Experts by Experience* (Cancer survivors and family caregivers); (ii) *Experts by Profession* (e.g., doctors, nurses, psychologists). Three individual workshops with representatives from both groups will be held in different locations across Ireland. A consensus workshop will be held online to collate results via a voting system and establish the design of an adapted group-based FCR intervention. The workshops will be co-facilitated by the lead researcher, a Patient and Public Involvement (PPI) partner and a member of the project steering committee.

**Discussion:**

Group-based interventions offer an efficient way to deliver targeted FCR support for young adult cancer survivors, while providing peer connection. The adapted peer-co-designed group-based FCR intervention will subsequently undergo feasibility evaluation to determine its acceptability and potential for broader implementation across Irish cancer centres.

## Introduction

Fear of cancer recurrence (FCR) is one of the most common unmet needs among cancer survivors, with approximately 59% reporting at least moderate levels of FCR and close to one in five patients experiencing high or clinically significant levels. ^1– 4^ This is pointedly higher for younger adult patients (84%) for a number of reasons, including their likely long survival rates and risk of developing secondary malignancies. ^5^ Early-onset cancer in young adults under 50 years has increased over the past two decades, creating distinct physical, psychosocial and support needs. ^6, 7^ Their experience is often socially isolating and marked by significant distress and uncertainty with attainment of life milestones such as education, career, relationships and family considerably impacted by FCR. ^6, 8, 9^ This highlights the growing need to tailor age-specific psychological support groups with peers of similar age. ^9, 10^

The effectiveness and efficacy of various psychological interventions for FCR have been widely demonstrated, particularly in a one-to-one format. FCR psychological interventions need to be accessible and efficient to meet the needs of the growing population of people living with and beyond cancer, with growing healthcare costs and limited resources. ^11^ Group-based approaches are more scalable, can maximise resources and provide peer support. ^12, 13^ While adapting evidence-based interventions for new contexts is regarded as more efficient than developing entirely new approaches, their effectiveness may diminish when they are replicated without modification. ^14^ Wide variation in existing group-based FCR programmes limits evidence on optimal design, highlighting the need for further research on how best to adapt one-to-one FCR interventions for different clinical settings, particularly for young adult cancer survivors.

Adapting existing interventions, in order to work in practice, need to be co-designed with both professional and PPI stakeholders to ensure they are based on the lived experiences of individuals living with and beyond cancer. ^15^ The focus of patient and public involvement (PPI) in clinical research has become increasingly essential in the past decade to ensure meaningful outcomes by empowering patients and the public to play an active role in their healthcare decisions and facilitate shared decision-making. ^16^ This study will use the ADAPT framework and co-design to adapt an existing contemporary FCR intervention, currently delivered in a one-to-one format, into a group setting for cancer survivors at a large comprehensive cancer centre in Ireland. ^14, 17^ The intervention is grounded in the theoretical framework of Acceptance and Commitment Therapy (ACT), incorporating core ACT processes to support psychological flexibility and behaviour change. ^11, 18– 20^ An initial feasibility study undertaken within the hospital suggested a strong interest in a group-based FCR intervention, highlighting the need for high-quality empirical research. ^21^ This study will focus on the design and delivery aspects that are imperative for the adaptation and implementation of intervention, appropriately tailored to the needs of young adult cancer survivors.

## Aims

To co-design the adaptation of a group-based intervention that is feasible and appropriate for young adult patients (aged 18–50 years) with at least moderate levels of FCR-related distress.

## Objectives

• To collaborate with people living with and beyond cancer, family caregivers and health professionals, in adapting an existing contemporary one-to-one FCR intervention to a group-based format that is age-specific.
• To ensure the logistics of the design are based on the lived experiences of individuals living with and beyond cancer through stakeholder partnership, while maintaining the core components of the psychological intervention, to ensure accessibility, uptake and sustained engagement.
• To co-design an appropriate management intervention for psychosocial distress across the spectrum of FCR needs, which are scalable and transferrable to psycho-oncology multidisciplinary teams recently established in other cancer centres nationally, and will be sustainable as a survivorship-friendly intervention.

## Methods

### ADAPT framework

This study is part of a larger project that aims to adapt and test an existing one-to-one FCR psychological intervention into a group setting. Table 1 outlines the steps in the ADAPT framework with details relating to this study. ^14^ This phase follows step 2 in the process.

**Table 1.** ADAPT framework for FORGE study.

ADAPT step	Key activities
*Step 1* Assess the rationale for intervention, and consider intervention-context fit	• Conducted a prevalence survey with the target population to identify different FCR levels (N = 394) • Conducted a scoping review on existing programmes • Selected the FCR psychological intervention for adaptation
*Step 2* Plan and undertake adaptations	• Establish an adaptation team (steering committee) comprising stakeholders with diverse expertise • Conduct co-design workshops with participants reflective of target population • Collate data from co-design workshops for intervention adaptation
*Step 3* Plan and undertake piloting and evaluation	• Plan to undertake feasibility pilot study • Evaluate adapted group-based intervention through survey and questionnaires
*Step 4* Implement and maintain the adapted intervention at scale	• N/A as this is a feasibility pilot study

### Intervention core components

The intervention selected for adaptation is an existing ACT-informed FCR programme currently delivered on an individual basis, for cancer survivors at a large comprehensive cancer centre in Ireland. The programme includes several evidence-based CBT components, including psychoeducation, cognitive restructuring, mindfulness, and worry management techniques ( Table 2). To maintain intervention fidelity, the therapeutic content and underlying behaviour change elements will remain unchanged throughout the co-design process. Instead, the co-design activities will focus on implementation and delivery considerations, including session logistics, group composition, and venue preferences. Participants will be informed that the intervention framework comprises a minimum of two hours of content delivered across two sessions, within which they can contribute to the design and optimisation of the programme’s format and delivery.

**Table 2.** Core Components of existing FCR Intervention

	Core Component
1.	Psychoeducation about FCR
2.	Cognitive restructuring
3.	Worry management
4.	Acceptance of uncertainty
5.	Values clarification
6.	Choice point decision making
7.	Mindfulness and grounding

### Patient and Public Involvement (PPI)

A PPI panel comprising three experts with lived experience of cancer and aged between 18–50 years, informed the development of this protocol. This process included the development of participant information leaflets, research questions, and practical considerations for the workshops. The PPI partners will co-facilitate the co-design workshops to create an open and supportive environment, enhance credibility and ensure more meaningful and participant-centred discussion. They are members of the project steering committee, together with representatives from psychology, nursing, medicine and relevant academic fields. The committee will inform and shape the overall project decision-making by drawing on their knowledge, experience and expertise to ensure the project achieves the intended outcomes and impact. The PPI panel contribution will be reported using the short form Guidance for Reporting Involvement of Patients and the Public (GRIPP2-SF). ^16^

### Co-design participants

This study will use the principles of co-design through stakeholder consultation to conduct three workshops with a minimum of ten participants recruited for each workshop. ^22, 23^ Participants will be **Experts by Experience** (Cancer survivors and family caregivers) and **Experts by Profession** (e.g., doctors, nurses, psychologists) to adapt an existing contemporary one-to-one FCR intervention, currently delivered in a large comprehensive cancer centre in Ireland, into the group-based format.

The workshops will be promoted via social media, patient cancer advocacy groups, and healthcare professional networks. All cancer types and healthcare professions will be encouraged to participate. Interested individuals can contact the lead researcher to ask questions about the study and receive the Participant Information Leaflet by post or email, or access the leaflet through a QR code on the poster. Written consent will be obtained in person at the workshop.

### Inclusion criteria

Cancer survivors ( **Experts by Experience**)

• Received a diagnosis of any cancer type and completed active cancer treatment at least one year previously (i.e. surgery, radiotherapy, chemotherapy).
• Aged between 18 and 50 years.
• Have experienced Fear of Cancer Recurrence or have a specific interest in the topic.
• Be able to provide informed written consent.
• Be fluent in English language.

Family Caregiver Representatives ( **Experts by Experience**)

• Be the family caregiver for someone who has completed active cancer treatment at least one year previously who were aged between 18 and 50 years at time of cancer treatment completion.
• Be the family caregiver for someone who has experienced Fear of Cancer Recurrence or have a specific interest in the topic.
• Be able to provide informed written consent.
• Be fluent in English language.

Healthcare Professionals ( **Experts by Profession**)

• Medical, nursing, psychologist, social worker, physiotherapist, occupational therapist or other healthcare professional working and involved in the care of patients, aged between 18 and 50 years, living with and beyond cancer.
• Be able to provide informed written consent.
• Be fluent in English language.

### Exclusion criteria

Cancer survivor/Family Caregiver Representatives

• Cancer patients with evidence of disease recurrence or metastases.
• Caring for someone with evidence of disease recurrence or metastases.
• Unable to provide informed consent.
• Not fluent in English language.

Healthcare Professionals

• Not working with cancer patients.

Table 3 outlines the proposed recruitment matrix.

**Table 3.** Proposed recruitment matrix

Characteristic	Cancer Survivors	Family Caregivers	Healthcare Professionals
Stakeholder group	Individuals with lived experience of cancer	Individuals with lived experience of supporting a family member with cancer	Professionals involved in cancer survivorship care
Age at treatment completion	Representation across 18–30, 31–40 and 41–50 years where feasible	Caring for survivors diagnosed/treated between 18–50 years	Professionals caring for adults aged 18–50 years
Sex/Gender	Diverse representation where possible	Diverse representation where possible	Diverse representation where possible
Cancer type	Range of cancer diagnoses where feasible	Experience supporting different cancer diagnoses	Experience caring for patients with varied cancer diagnoses
Time since treatment completion	1–2 years, 3–5 years, >5 years	Caring experience relating to different survivorship stages	Varying years of professional experience
Geographic location	Urban and rural settings	Urban and rural settings	Hospital and community settings where applicable
Previous experience of FCR	Range of FCR experiences and concerns	Experience supporting FCR concerns in a family member	Experience with cancer survivors in clinical practice
Professional/educational background	Diverse occupational backgrounds	Diverse occupational backgrounds	Nursing, medicine, psychology, social work, physiotherapy, occupational therapy, and other relevant disciplines

### Study procedures

This study will use a modified Nominal Group Technique (NGT) through a series of workshops. NGT is a highly structured, face-to-face group intervention that poses specific questions for discussion, empowering participants by giving them an opportunity to have their voices and opinions heard by all members. ^24^ The group workshop is highly structured and involves two to three questions which are sent to the participants in advance:

1. What would be the most effective way to deliver the group-based programme? (i.e., number/frequency/length of sessions)
2. What age groups/cancers would be best together at the group-based programme? (i.e., specific age groups/mixture of ages, different/same cancers)
3. What would be the preferred venue for the group-based programme? (i.e., hospital/cancer support centre, virtual)

The NGT method is highly adaptive, and variations can be influenced by a number of factors, including consensus or generalisability required for the topic. ^25^ Typically, NGTs involve a single workshop; however, in this study, there will be three individual face-to-face workshops held in different geographically located areas to ensure broad national representation, encompassing both rural and urban areas in Ireland. Participants will be invited to attend one workshop to discuss the first three steps (i.e. idea generation, sharing ideas, and refining ideas). Each workshop will follow the same format posing the same questions to each group. The final step of ranking and voting on ideas to reach consensus will be held virtually, with participants from all three workshops invited to participate. ^26^

While NGTs typically range from two to fourteen participants, a maximum of seven has been recommended. ^25^ It is anticipated there will be a minimum of ten participants recruited in this study for each of the three workshops – six to seven cancer survivors/family carer representatives ( **Experts by Experience)** and three to four healthcare professionals ( **Experts by Profession**). This approach is to ensure that the different participant age ranges (18–50 years) are represented and allows for a variety of healthcare professional representation. This number will also allow for attrition while ensuring representation of each cohort. Each workshop will be facilitated by the lead researcher and a member of the steering committee, including a PPI partner.

Following an introduction to the study and purpose of the workshop by the lead researcher, participants will be divided into subgroups of three or four with a mix of Experts by Experience and Experts by Profession in each group. This approach is a modification to the NGT structure to combine steps one and two (‘idea generation’ and ‘sharing of ideas’) to allow for the larger group structure and time constraints, while ensuring each participant has an opportunity to contribute. ^25^ Each subgroup will be facilitated by a member of the research team. The lead researcher will explain the discussion question and allow 30–40 minutes for each subgroup to generate suggestions. Post-it notepads will be used to colour code answers from each question. A final discussion will take place with the whole group to clarify and refine the ideas generated.

All participants from each of these three workshops will then be invited to attend a virtual stakeholder meeting to complete the final stage of the co-design process- ‘ranking/voting preference of ideas’. The ideas generated from the three workshops will be compiled and presented to the group. The number of votes for each idea will be added up to reach a final consensus. Results will be available in real time, with participants seeing them for each question. ^27^

### Data collection

Participants will provide demographic details at the beginning of the face-to-face workshops to outline representation from both Experts by Experience and Experts by Profession. The workshops will not be recorded but written post-it notes generated during the workshops will be secured by the lead researcher.

## Results

### Data analysis

Written responses generated during the three workshops will be collated and analysed using summative content analysis. Responses will first be grouped according to each workshop question. Similar suggestions arising within and across workshops will be combined into broader categories through discussion among the research team, while maintaining the original meaning of participants’ contributions. The frequency and recurrence of ideas across workshops will be used to identify common priorities and inform the development of a consolidated list of intervention design options for presentation at the final consensus workshop.

All participants from the three workshops will then be invited to attend a virtual stakeholder meeting to complete the final consensus stage of the co-design process. Following synthesis of the workshop findings, a consolidated list of intervention design options for each discussion question will be presented to participants. Participants will vote for their preferred option using Mentimeter, a third-party platform specialising in online interactive voting. ^28, 29^ Votes will be anonymously submitted and displayed in real time to the group. The number of votes received for each option will be combined, and those receiving the greatest level of support will inform the final intervention design. This simplified voting approach was selected to minimise participant burden while ensuring all stakeholder perspectives contributed to the final intervention design. Where there is no clear preference between options, additional discussion will be facilitated before a further round of voting is undertaken.

## Discussion

Group-based interventions offer a structured and efficient approach to delivering targeted FCR treatments while fostering a peer support environment. However, variations in current group design highlight the need for further work to adapt an existing contemporary group-based FCR intervention that will address the needs of young adult cancer survivors experiencing at least moderate levels of FCR in Ireland. The outcome of this study will provide the structure for a psychological intervention that has been created by peers who have experienced the same condition and collaborated on its design. The next phase of the overall project (ADAPT Step 3) will involve a feasibility pilot to assess the suitability and acceptability of the adapted, scalable group-based FCR intervention for use across cancer centres in Ireland.

## Ethical approval

This research has been approved by SJH/TUH Joint Research Ethics Committee (St James’s Hospital/Tallaght Hospital) on 6 ^th^ May 2025, Project ID 4347. Written Informed consent will be obtained by all participants.

## Data Availability

No data are associated with this article.
