# Supplementary material for: Fear Of cancer Recurrence Group-based intervEntion (FORGE): Study protocol for the co-design of a psychological intervention for young adult cancer survivors
Source: HRB Open Res. 2026 Nov 12;9:63. Originally published 2026 Jun 16. [Version 4] doi: 10.12688/hrbopenres.14446.4 (PMC13680839; doi:10.12688/hrbopenres.14446.4)
Supplement: Peer review history

# Reviewer report: Reviewer response for version 2

**DOI:** 10.21956/hrbopenres.16040.r58272

**Published:** 2026-10-19

**Authors:** Vijaya Prasad Barre

**Affiliations:** 1 All India Institute of Medical Sciences (AIIMS), New Delhi, Delhi, India

This protocol describes the co-design of a group-based psychological intervention for fear of cancer recurrence among young adult cancer survivors in Ireland. The use of the ADAPT framework and involvement of cancer survivors, family caregivers, and healthcare professionals are strengths. The authors should clarify the core components of the existing intervention and identify which aspects will be retained or adapted. Further details on family caregivers and health professionals representation, synthesis of workshop findings, and voting and consensus procedures would strengthen methodological clarity.

 Approved with Reservations: The study addresses an important issue in psycho-oncology. Clarification of the intervention adaptation and consensus procedures would strengthen the manuscript without requiring changes to its fundamental design.

Is the study design appropriate for the research question?

Yes

Is the rationale for, and objectives of, the study clearly described?

Yes

Are sufficient details of the methods provided to allow replication by others?

Partly

Are the datasets clearly presented in a useable and accessible format?

Yes

Reviewer Expertise:

Psycho-oncological research

I confirm that I have read this submission and believe that I have an appropriate level of expertise to confirm that it is of an acceptable scientific standard, however I have significant reservations, as outlined above.

# Response

**Published:** 2026-11-05

**Authors:** Catherine O'Brien

**Affiliations:** School of Surgery, Trinity College Dublin School of Medicine, Dublin, Leinster, Ireland

**Thank you for taking the time to review our protocol. We are grateful for your valuable feedback and suggestions. Some of the recommendations had previously been made in the most recent version 3 in response to Reviewer 2, which was not published at the time of your review.**

***Comment 1:       The authors should clarify the core components of the existing intervention and identify which aspects will be retained or adapted.***

**Intervention core components (previously amended in version 3)**

 The intervention selected for adaptation is an existing ACT-informed FCR programme currently delivered on an individual basis, for cancer survivors at a large comprehensive cancer centre in Ireland. The programme includes several evidence-based CBT components, including psychoeducation, cognitive restructuring, mindfulness, and worry management techniques (Table 2). To maintain intervention fidelity, the therapeutic content and underlying behaviour change elements will remain unchanged throughout the co-design process. Instead, the co-design activities will focus on implementation and delivery considerations, including session logistics, group composition, and venue preferences. Participants will be informed that the intervention framework comprises a minimum of two hours of content delivered across two sessions, within which they can contribute to the design and optimisation of the programme's format and delivery.

***Comment 2:       Further details on family caregivers and health professionals representation, synthesis of workshop findings, and voting and consensus procedures would strengthen methodological clarity.***

**Family caregivers and healthcare professional representation (previously updated in version 3)**

 Table 3 outlines the proposed recruitment matrix for family caregivers and healthcare professionals.

**Synthesis of workshop findings & Consensus workshop procedures (amended for version 4)**

**Data analysis**

 Written responses generated during the three workshops will be collated and analysed using summative content analysis. Responses will first be grouped according to each workshop question. Similar suggestions arising within and across workshops will be combined into broader categories through discussion among the research team, while maintaining the original meaning of participants' contributions. The frequency and recurrence of ideas across workshops will be used to identify common priorities and inform the development of a consolidated list of intervention design options for presentation at the final consensus workshop.

 All participants from the three workshops will be invited to attend a virtual stakeholder meeting to complete the final consensus stage of the co-design process. Following synthesis of the workshop findings, a consolidated list of intervention design options for each discussion question will be presented to participants. Participants will vote for their preferred option using Mentimeter. Votes will be anonymously submitted and displayed in real time to the group. The number of votes received for each option will be combined, and those receiving the greatest level of support will inform the final intervention design. This simplified voting approach was selected to minimise participant burden while ensuring all stakeholder perspectives contributed to the final intervention design. Where there is no clear preference between options, additional discussion will be facilitated before a further round of voting is undertaken.

# Reviewer report: Reviewer response for version 2

**DOI:** 10.21956/hrbopenres.16040.r58140

**Published:** 2026-09-26

**Authors:** Jan Christopher Cwik

**Affiliations:** 1 Hochschule Niederrhein, Krefeld, Germany

This protocol describes the co-design of a group-based fear of cancer recurrence intervention for young adult cancer survivors in Ireland. The proposed combination of three regional workshops and a subsequent online prioritization meeting is well suited to exploring stakeholder preferences for delivery, group composition, and setting.

 Involving three PPI partners in protocol development, workshop facilitation, and the steering committee is a strength. Identifying Acceptance and Commitment Therapy as the theoretical foundation, including explicit workshop questions, and distinguishing this development phase from subsequent feasibility testing are also helpful.

 My assessment concerns whether the proposed co-design process is sufficiently specified to produce a transparent and usable intervention adaptation. This protocol does not require clinical effectiveness results, a randomized comparison, or a trial-style power calculation. However, the starting intervention, how stakeholder contributions are handled, and the rules linking voting to final design decisions need greater operational clarity.

 Major aspects for a potential revision

 1. Identify the source intervention and distinguish its core components from the features open to adaptation.

 The Introduction now identifies ACT as the theoretical foundation, which addresses part of the earlier reviewer’s question. However, the specific one-to-one intervention remains described primarily by its setting rather than by an identifiable program or treatment specification.

 Please identify the intervention’s name, source or manual, current session structure, intended recipients, and principal therapeutic components. If this is a locally developed ACT-informed program rather than a named intervention with its own evaluation, state that explicitly. Distinguish evidence supporting ACT-based approaches more generally from evidence for this particular program.

 The objective of maintaining core components is appropriate, but you need to identify those components. A concise table could distinguish the intervention’s therapeutic functions, its current individual-delivery format, and the features that participants may recommend changing.

 This matters because the three workshop questions focus mainly on session logistics, group composition, and venue. Please clarify whether this study is deliberately limited to co-designing delivery arrangements around fixed therapeutic content, or whether participants can also influence materials, exercises, language, and the way therapeutic processes operate in a group.

 Neither scope is inherently inappropriate. However, changing the number or duration of sessions may affect the content that can be delivered, so the protocol should explain how it will address these interdependencies.

 Please also describe what information participants will receive about the existing intervention before making recommendations. Identify the intended output of this phase—for example, a documented delivery specification and adaptation record—and who will check that the proposed format remains consistent with the intervention’s therapeutic rationale. A final adapted manual cannot be expected before co-design, but the process for producing it should be clear.

 2. Align stakeholder recruitment with the intended intervention population while preserving appropriate diversity.

 The aim specifies patients aged 18–50 years with at least moderate FCR-related distress. Workshop eligibility is broader: survivors may participate if they have experienced FCR or simply have a specific interest in the topic.

 Broad participation can be valuable in co-design, and I would not require every contributor to meet a clinical FCR threshold. Nevertheless, the current criteria do not ensure that the people the intervention is intended to serve will be adequately represented.

 Please describe how the recruitment process will secure meaningful participation by survivors with relevant FCR experience, including those who have experienced appreciable distress. Clarify whether severity or experience will be characterized through self-report, a brief measure, or another procedure. This information need not function as an exclusion rule.

 The proposed six to seven Experts by Experience combine survivors and caregivers. Please specify the intended balance within that group rather than treating their perspectives as interchangeable. Explain whether survivor–caregiver pairs can participate together and how people who qualify under more than one stakeholder category will be counted.

 A pragmatic recruitment matrix would help describe the intended diversity across age, cancer experience, time since treatment, geography, and professional background. It need not impose statistically representative quotas.

 Several eligibility details also warrant clarification. For survivors, the age criterion appears to refer to current age; for caregivers, it refers to the survivor’s age at treatment completion. Please explain whether this difference is intentional. Justify the study-specific 18–50-year range and the requirement that active treatment ended at least one year previously, and clarify how ongoing maintenance or endocrine treatment will be handled.

 Finally, distinguish eligibility for this co-design phase from intended eligibility for the later intervention. Findings from the stated recurrence-free, non-metastatic, English-speaking participant group should not automatically be generalized to all people living with and beyond cancer.

 3. Explain how the modified NGT will preserve individual contributions in mixed-status groups.

 Dividing each workshop into subgroups of three or four is a reasonable practical response to the planned overall attendance. I do not regard recruiting approximately ten participants per workshop as inherently problematic.

 The more important modification is combining idea generation and sharing within mixed groups of survivors, caregivers, and professionals. This could make it difficult to distinguish independently held preferences from suggestions developed after hearing more influential participants.

 Please describe the procedures intended to support balanced participation. These might include a brief period of individual written idea generation, structured opportunities for each person to contribute, and an explicit invitation to record disagreement or propose alternatives. An equivalent approach is acceptable; the key is to explain how you will retain less vocal participants’ views.

 Please also clarify whether participants may be grouped with their own treating professionals or family members, and how you will manage potential constraints on speaking openly. Mixed groups can facilitate useful exchange, but do not assume they will eliminate differences in authority or confidence.

 The PPI co-facilitation role is welcome. Specify the number and roles of facilitators needed for simultaneous subgroups, their preparation, and how you will apply the same procedure across the three locations. Distinguish facilitators’ contributions from participant-generated ideas.

 An indicative agenda should show whether the stated 30–40 minutes applies to each question and how clarification, breaks, and whole-group discussion fit within the total workshop duration.

 4. Describe how written workshop material will be converted into the final voting options.

 The decision not to audio-record the workshops is not itself a methodological flaw. Written outputs may be sufficient for this focused prioritization task. However, the protocol should explain how it will retain enough context to interpret those outputs accurately.

 The current description of summative content analysis focuses on identifying recurring keywords and common suggestions. Keyword recurrence alone may not preserve meaning. For example, two notes mentioning virtual delivery could express opposing preferences or identify different conditions under which that format would be acceptable.

 Please specify the unit of analysis, who will categorize the material, how ambiguous statements will be clarified, and how similar or overlapping suggestions will be merged. Describe how original notes will remain traceable to the consolidated options presented for voting.

 Clarify whether facilitators will document the reasons, qualifications, and disagreements expressed during the final discussion. Where no such record is planned, acknowledge that the analysis concerns the written suggestions rather than a comprehensive account of the discussion.

 Also distinguish how often an idea appears on Post-it notes from how many individual participants support it. A jointly produced subgroup note does not necessarily represent agreement by every subgroup member.

 Please explain whether all distinct suggestions will reach the voting stage or whether some will be screened out beforehand. Any screening criteria should be transparent and should not automatically remove an accessibility concern merely because it was mentioned infrequently.

 Finally, describe how participants, including the PPI partners, will be able to check that the consolidated options accurately represent the original contributions before voting begins.

 5. Specify the voting procedure, the meaning of consensus, and the authority for final decisions.

 The statement that participants will rank each idea from 1 to 5 is ambiguous. Please clarify whether participants will independently rate every option on a five-point scale, select and rank their five preferred options, or use another procedure. State how unselected options, ties, incomplete ballots, and abstentions will be handled.

 The protocol should then explain the decision rule. Adding votes or points provides a prioritization result, but readers still need to know what qualifies a proposed feature for inclusion in the adapted program and what the authors mean by consensus.

 A numerical agreement threshold is not mandatory for every NGT study. However, include a transparent rule for interpreting the results, resolving tied or incompatible preferences, and documenting disagreement. If the intended output is a ranked set of stakeholder priorities rather than a formally agreed consensus, describe it accordingly.

 Please consider reporting both the strength of preference and the breadth of support where relevant to the chosen voting system. The analysis across multiple groups discussed in the manuscript’s reference 25 is particularly relevant to this distinction.

 All regional workshop participants will be invited to the final online meeting, but not all may attend. Specify the minimum participation or stakeholder coverage considered sufficient, how differential attendance will be described, and whether an equivalent voting opportunity will be available to those unable to join synchronously. Any alternative route should use the same information and avoid duplicate voting.

 Clarify whether votes will be confidential and whether aggregate results will be displayed only after the initial ballot has closed. The current reference to real-time results does not establish this sequence.

 Finally, explain how the voting results will interact with the steering committee’s clinical and practical judgments. Who has final decision-making authority? How will recommendations that conflict with therapeutic requirements or available resources be handled? Documenting these decisions, their reasons, and PPI involvement is preferable to presenting every final design feature as a direct product of consensus.

 6. Add proportionate safeguards and a data-management plan for the co-design activities.

 Ethical approval and the intention to obtain written consent are reported. Nevertheless, the protocol should briefly describe the practical safeguards for discussing potentially distressing cancer experiences in mixed groups.

 Please state how participants can pause, decline a discussion topic, or leave a workshop, and what support or referral arrangements will be available if participation becomes distressing. Distinguish the co-design activity from psychological treatment so that expectations are clear.

 Explain the confidentiality arrangements for group discussions, including the limits of what researchers can guarantee concerning information heard by other participants. The consent materials should also describe how withdrawal will operate once individual suggestions have been incorporated into group outputs.

 For the written notes, demographic information, and Mentimeter responses, identify what will be retained, whether responses will be identifiable, who will have access, and how data will be stored securely. Clarify the information that will be entered into the third-party voting platform and how the resulting dataset will be exported and managed.

 The statement that no underlying data accompany a protocol is appropriate. It would nevertheless be useful to describe which non-identifying materials and outputs will subsequently be made available, such as workshop guides, the coding framework, aggregated voting results, and the adaptation record.

 Minor aspects for a potential revision

 1. Report study status and distinguish planned procedures from completed activities.

 Ethical approval is dated May 2025, and Table 1 describes the prevalence survey and scoping review as completed. Please provide the recruitment/workshop timetable and indicate which activities had begun or been completed when you prepared this protocol version.

 This is especially relevant to the voting and synthesis rules: readers should be able to distinguish procedures specified before data collection from amendments introduced later. If workshops have already occurred, report that transparently rather than retrospectively describing new procedures as prespecified.

 A brief indication of where dated protocol amendments will be documented would be sufficient.

 2. Clarify access arrangements and moderate claims of national representation.

 Holding workshops in three regions can broaden geographical participation, but it does not by itself ensure national representativeness. Describe the intended regional coverage and use language such as geographically diverse participation unless stronger representativeness claims can be supported.

 Please state whether travel expenses, reimbursement for participants’ time, accessible venues, flexible scheduling, or technical support for the online meeting are available. These practical details are directly relevant to a project concerned with accessible intervention design.

 Where participants cannot attend in person or use the online platform, describe any available accommodation and acknowledge the remaining limits of recruitment through the stated channels.

 3. Correct the source attribution and presentation issues.

 The statement that a local feasibility study indicated interest in group delivery cites reference 21, which is a methodological paper defining feasibility and pilot studies rather than a report of that local work. Please cite the relevant study or identify the finding as unpublished local work, with enough information to understand its basis. Clarify its relationship, if any, to the 394-person survey in Table 1.

 The planned GRIPP2-SF reporting is appropriate, but reference 16 is a meta-epidemiological evaluation of PPI reporting. Please also cite the original GRIPP2 reporting checklist publication.

 The Discussion refers to adapting an existing group-based intervention, whereas the rest of the protocol describes adapting a one-to-one intervention into groups. Please correct this distinction. In Table 1, align the explanation that Step 4 is not applicable because this is a feasibility pilot with the current co-design phase and the wider project.

 Move the planned data analysis out of the otherwise empty Results heading. Describe NGT as a structured consensus or prioritization method rather than a therapeutic group intervention.

 4. Keep the claims proportionate to what co-design can establish.

 The rationale for potential peer support, accessibility, and resource efficiency is understandable. However, these should remain anticipated advantages to be evaluated, not benefits established by this protocol.

 Similarly, stakeholder judgments about the “most effective” way to deliver the program concern perceived suitability and preferences, not comparative clinical effectiveness. Consider wording the first workshop question around acceptable and workable delivery arrangements while retaining space for participants to explain their reasoning.

 Keep the distinction between this phase and the later feasibility pilot explicit throughout. Co-design can produce a stakeholder-informed adaptation; feasibility, sustained engagement, clinical benefit, and resource efficiency require subsequent evaluation.

Is the study design appropriate for the research question?

Yes

Is the rationale for, and objectives of, the study clearly described?

Yes

Are sufficient details of the methods provided to allow replication by others?

Partly

Are the datasets clearly presented in a useable and accessible format?

Yes

Reviewer Expertise:

Psycho-oncology; Psychotherapy; Clinical Psychology

I confirm that I have read this submission and believe that I have an appropriate level of expertise to confirm that it is of an acceptable scientific standard, however I have significant reservations, as outlined above.

# Response

**Published:** 2026-10-10

**Authors:** Catherine O'Brien

**Affiliations:** School of Surgery, Trinity College Dublin School of Medicine, Dublin, Leinster, Ireland

We are grateful to the reviewer for their detailed and constructive feedback. A number of revisions have been made to improve clarity and methodological transparency. While several suggestions would undoubtedly strengthen the reporting of the completed study, we considered some of the requested detail to extend beyond the purpose of a protocol manuscript, as many of these decisions and outputs will emerge through the co-design process itself. We have therefore sought to provide sufficient methodological detail while maintaining the flexibility necessary for meaningful stakeholder engagement.

 Comment 1: Source intervention and intervention components

 We thank the reviewer for this helpful suggestion. The manuscript has been revised to provide additional information on the existing intervention, including its ACT-informed theoretical foundation and key CBT components. As this manuscript presents a protocol, our intention is to outline the intervention sufficiently to contextualise the proposed co-design process, while recognising that a detailed intervention specification will be developed and reported following completion of the co-design phase.

 Comment 2: Stakeholder recruitment and representation

 We appreciate the reviewer's thoughtful comments regarding participant representation. Clarifications have been added regarding eligibility criteria and stakeholder groups. Given the aims of the co-design process, a purposive approach will be used to ensure a range of perspectives is represented. While we acknowledge the value of a more detailed recruitment framework, we considered this level of specification beyond the scope of the current protocol.

 Comment 3: Modified NGT process

 We thank the reviewer for highlighting the importance of ensuring equitable participation across stakeholder groups. Additional detail has been provided regarding workshop facilitation and the planned modified nominal group technique. As the protocol describes a flexible co-design process, some procedural elements will necessarily be refined in response to participant engagement and workshop discussions.

 Comment 4: Development of voting options

 We appreciate this suggestion. Given that the recommendations will be generated through the co-design process itself, the precise procedures for categorisation and refinement will be reported in the findings arising from the completed study rather than within the protocol.

 Comment 5: Consensus and voting procedures

 We thank the reviewer for this observation. The purpose of the voting exercise is to identify stakeholder priorities and guide adaptation decisions rather than to achieve formal consensus. We have therefore outlined the planned prioritisation process while retaining flexibility for decisions that will be informed by participant contributions during the workshops.

 Comment 6: Safeguards and data management

 We appreciate the reviewer's attention to participant welfare and study governance. Ethical oversight, consent procedures, and data management processes are described within the protocol. More detailed operational procedures will be implemented in accordance with institutional and ethical requirements and were considered outside the intended scope of this protocol manuscript.

 Minor Comment 1: Study status

 We thank the reviewer for this helpful recommendation. Additional clarification has been included to distinguish the current protocol from the broader programme of work and to clearly outline the focus of the present study.

 Minor Comment 2: Representativeness and accessibility

 We appreciate this comment and have revised the wording to avoid implying national representativeness. Consistent with co-design methodology, the aim is to engage a range of relevant perspectives rather than achieve a representative sample.

 Minor Comment 3: Presentation and clarity

 We thank the reviewer for these observations. The manuscript has been reviewed and amended where appropriate to enhance clarity and consistency.

 Minor Comment 4: Strength of claims

 We appreciate this observation and have reviewed and amended where appropriate to ensure the claims remain proportionate to the aims of the proposed co-design study. The effectiveness of the adapted intervention will be explored in future phases of research.

# Reviewer report: Reviewer response for version 1

**DOI:** 10.21956/hrbopenres.15918.r57181

**Published:** 2026-08-12

**Authors:** Johanne D. Lyhne

**Affiliations:** 1 University Hospital of Southern Denmark, Beriderbakken, Denmark

Great and important study, and lovely to see you prioritizing this important step.  

 Comments: 

 Please state if it is a specific intervention you aim to adapt - "an existing one-to-one FCR psychological intervention". Can we get more information? Is it an evidence-based intervention? How many session? What theoretical content? 

 Please state if you aim for an equal distribution between experts by experience and experts by profession in the workshops. 

 Please state if you will be selective/inclusive regarding cancer type. Will you aim for a broad representation of diagnoses? 

 Please state if you will be selective/inclusive regarding professions. Will you aim for a broad representations of clinical staff? 

 Consider the possibility to include experts by experience who are also experienced with FCR. If you design an intervention for survivors with at least moderate FCR, consider use this as an inclusion criteria for this process as well. 

 Do you have any considerations regarding virtual delivery? 

 Good luck!

Is the study design appropriate for the research question?

Yes

Is the rationale for, and objectives of, the study clearly described?

Yes

Are sufficient details of the methods provided to allow replication by others?

Partly

Are the datasets clearly presented in a useable and accessible format?

Not applicable

Reviewer Expertise:

Psycho-oncology

I confirm that I have read this submission and believe that I have an appropriate level of expertise to confirm that it is of an acceptable scientific standard.
